# Urinary lipid profile of patients with coronavirus diseases 2019

**DOI:** 10.3389/fmed.2022.941563

**Published:** 2022-09-26

**Authors:** Misato Kida, Tatsuro Nakamura, Koji Kobayashi, Tatsuo Shimosawa, Takahisa Murata

**Affiliations:** ^1^Department of Animal Radiology, Graduate School of Agricultural and Life Sciences, The University of Tokyo, Tokyo, Japan; ^2^Department of Clinical Laboratory, International University of Health and Welfare, Chiba, Japan

**Keywords:** COVID-19, lipidomics, proinflammatory lipid mediators, metabolites, urine analysis

## Abstract

The coronavirus diseases 2019 (COVID-19) pandemic caused by severe acute respiratory syndrome coronavirus 2 (SARS-CoV-2) is ongoing. Over 490 million people have been infected with this virus worldwide. Although many patients present with lower respiratory symptoms, some may progress to acute respiratory distress syndrome and even multi-organ damage. Therefore, there is an urgent need to establish treatment and management methods for this infectious disease. Here, we comprehensively analyzed urinary lipid mediators and their metabolites to identify non-invasive biomarkers that reflect the disease status of COVID-19 patients. We diagnosed 16 patients by polymerase chain reaction (PCR) analysis, who presented with mild-to-moderate symptoms, including fever and cough, between May and October 2020 in Japan, and collected their urine samples. Using mass spectrometry, we analyzed the lipid metabolites in these urine samples. In all the urine samples from the patients, 21 types of fatty acids and their metabolites were consistently detected in the samples among the 214 metabolites which were analyzed. Interestingly, urinary levels of fatty acids, docosahexaenoic acid was increased by approximately 3-fold in patients with COVID-19 compared to those in healthy subjects. Metabolites of major proinflammatory lipid mediators, PGE_2_, TXA_2_, and PGF_2α_, were also detected at significantly higher levels in the urine of patients with COVID-19. These observations suggest that urinary lipids can reflect the inflammatory status of patients with COVID-19, which can be a useful index to manage this disease.

## Introduction

Coronavirus disease 2019 (COVID-19) is an infectious disease caused by the severe acute respiratory syndrome coronavirus 2 (SARS-CoV-2). Despite initial efforts to control its spread, the disease soon became a pandemic and has since spread to numerous countries worldwide. This pandemic has presented an unprecedented threat to global public health. The predominant symptoms of COVID-19 occur in the respiratory system. Fever, cough, taste/smell disorders, and respiratory difficulties are the most common complaints (1, 2). Although most COVID-19 patients experience only mild-to-moderate symptoms, the disease status can rapidly progress to severe, particularly in the absence of adequate medical care. Severely ill individuals present with life-threatening acute respiratory distress syndrome.

Coronavirus diseases 2019 is a heterogeneous disease characterized by diffuse alveolar damage, endothelial damage, thrombosis of small and medium-sized vessels, pulmonary embolism, and inflammatory cell infiltration (3, 4). An uncontrolled systemic inflammatory response, known as a cytokine storm, also occurs. Cytokine storms are a result of the release of proinflammatory cytokines such as tumor necrosis factor-α, interleukin (IL)-1β, and IL-6 (5).

Lipid metabolism is also disturbed in COVID-19 patients (6). Bioactive lipids (lipid mediators) are involved in infectious and inflammatory responses by modulating leukocyte recruitment, platelet aggregation, and thrombus formation (7–10). These bioactive molecules are derived from polyunsaturated fatty acids (PUFA), such as arachidonic acid (AA), eicosapentaenoic acid (EPA), and docosahexaenoic acid (DHA), by enzymatic or non-enzymatic oxidation. Since the production and metabolism of lipid mediators vary with the type, stage, and tissue site of diseases (11, 12), clarification of the lipid mediator profile is useful for elucidating the pathophysiology and identifying biomarkers. Archambault et al. detected abundant production of bioactive lipids in bronchoalveolar lavage fluids (BALFs) from patients with severe COVID-19, suggesting that lipid metabolites can be useful indices of COVID-19 symptoms (13). Barberis et al. also reported an elevation in fatty acid levels in a large-scale plasma analysis of COVID-19 patients (14). Benjamin. S. et al. showed that systemic lipidomics changes in plasma distinguish COVID-19 disease severity (moderate to severe) from healthy subjects (15). However, since the sampling of BALFs and plasma requires medical treatment and care, these procedures lack versability.

We have focused on bioactive lipids and their metabolites excreted into body fluids, including urine, which can be sampled from patients even in non-medical facilities. In addition, the non-invasive urine collection does not stimulate an inflammatory reaction accompanied with lipid production. Thus, urine is a convenient and appropriate sample to observe the lipid production and body condition. In the present study, we attempted to determine the urinary lipid profiles of COVID-19 patients using lipidomics technology. We successfully detected changes in the levels of some fatty acids and lipid metabolites in the urine of patients with mild-to-moderate COVID-19.

## Materials and methods

### Sample information

Healthy subjects (male, 5, female, 11; mean age, 51.6 ± 3.2 years; range, 33–76 years) who have no symptoms and sixteen patients (male, 12, female, 4; mean age, 50.1 ± 13.2 years; range, 26–75 years) who were diagnosed with COVID-19 by reverse transcription polymerase chain reaction (RT-PCR) test in the Narita Hospital of International University of Health and Welfare, Japan were enrolled. These patients represented some symptoms such as fever and cough. They had respiratory failure requiring oxygen supplementation but not mechanical or non-invasive ventilation. We defined them as mild-moderate patients. Spontaneous voiding urine were collected immediately after diagnosis of COVID-19. They had not been treated with corticosteroids and non-steroidal anti-inflammatory drugs which inhibit lipid metabolism directly or indirectly in the 2 weeks before the urine and blood collection. The study protocol was approved by the Ethics Committee of the International University of Health and Welfare (20Nr-001) and the University of Tokyo (No. 10586-1). All experiments were performed in accordance with the approved guidelines. The symptom and information of each subject are described in Table 1.

**TABLE 1:** Clinical profiles of the study subjects.

	Healthy subjects	COVID-19 patients
Sex (No)	Male 5, Female 11, Total 16	Male 12, Female 4, Total 16
Age (year)	Mean 51.6 ± 3.2, Range 33–76	Mean 50.1 ± 13.2, Range 26–75
Symptom	None	Fever 15, Cough 12, Taste disorder 2, Smell disorder 1, Headache 3, Fatigue 6, Dyspnea 2, Diarrhea 3, Chest pain 2, Vomiting 1.

### Data processing

For analyzing the data of patients with multiple sampling, we selected the data which had the highest urinary level of tetranor-PGEM, the metabolites of prostaglandin (PG) E_2_ (PGE_2_) except for the co-relationship analysis.

### Blood chemical test

Venous blood was corrected into EDTA 2Na containing tube and centrifuged (1500G 10 min) to separate plasma. Plasma was kept −80 degree until the analysis. All the blood markers are measured automatically according to the manufacture’s protocol. Plasma C-reactive protein (CRP) and ferritin were measured by TBA-FX8 (Canon, Tokyo Japan) with Acuras-auto CRP-N and FER-LatexRX Seiken, respectively. D-dimer was measured by Cobas t711 with t-system hexamate d-dimer and procalcitonin were measured by Cobas e801 with elecsys brahmsPCT.

### Comprehensive analysis of lipid metabolites

Comprehensive analysis of lipid metabolites was performed by using a software method package [LC-MS/MS (8060)] for lipid mediators (Shimadzu, Kyoto, Japan) according to the manufacturer’s instructions. Data were analyzed by LabSolutions LCMS Ver. 5.65 (Shimadzu, Japan), as described previously (16). In brief, urine samples from patients or healthy subjects were collected and immediately diluted to 50% methanol. Urine samples were stored at −80°C until analysis. The samples (200 μl) were mixed with 0.05% formic acid (300 μl) and internal standard (10 μl) solution (Supplementary Table 1). The mixed solutions were loaded on solid phase extraction cartridges (OASIS HLB μElute, Waters, Milford, MA, USA) and washed with distilled water and hexane, then eluted with methanol (>99.8%, 100 μl) and injected into LCMS-TQ8060 (Shimadzu). Urinary creatinine concentrations were measured with a kit (Wako, Tokyo, Japan).

### Statistics

Data are expressed as mean ± SEM except for the age of healthy subjects and patients, which is expressed by mean ± SD. Statistical analyses were performed using Bell Curve for Excel 2015 software (Social Survey Research Information Co., Ltd. Tokyo, Japan). Statistical differences were analyzed by Student’s *t*-test. The correlation with the plasma biomarkers were analyzed *via* Pearson correlation analysis. *P* < 0.05 suggested that the difference in data was statistically significant.

## Results

### Patients: Diagnosis and blood test results

Sixteen patients were diagnosed for COVID-19. The patients presented with symptoms, including fever and cough, and their urine samples were collected between June and October 2020. The clinical characteristics of these patients are presented in Table 1. The patients included in this study were from Chiba and Tokyo Prefectures, Japan. We also recruited 16 healthy adults and collected their urine samples as controls. The data of the blood chemical test is shown in Table 2.

**TABLE 2:** The blood chemical test data.

No	CRP (mg/dl)	D-dimer (μg/dl)	Procalcitonin (ng/ml)	Ferritin (ng/ml)
1	14.59	n.d.	n.d.	n.d.
2	0.28	0.67	0.04	448
3	4.01	0.71	0.04	1183
4	0.82	0.4	0.06	199
5	1.08	1.22	0.02	404
6	3.84	1.38	0.2	318
7	3.5	1.94	0.06	541
8	0.83	0.62	0.03	322
9	0.82	0.4	0.03	143
10	0.3	0.75	0.03	502
11	1.25	3.02	0.02	227
12	0.44	1.42	0.04	935
13	1.08	0.88	0.05	79
14	0.58	0.41	0.04	292
15	4.89	0.55	0.05	310
16	1.99	2.12	0.13	1282
Mean ± SEM	2.51 ± 0.88	1.09 ± 0.19	0.05 ± 0.01	479.00 ± 94.82

### Urinary lipid profile of coronavirus diseases 2019 patients and healthy subjects

Using mass spectrometry, we comprehensively analyzed urine sampled from COVID-19 patients. The urinary lipid levels for individuals are listed in Supplementary Table 2. In all urine samples from these patients and the control group, we found that 21 types of lipids were consistently detected from the analyzed metabolites (Table 3 and mapping in Supplementary Figure 1). Among them, 14 were arachidonic acid (AA)-derived and three were linoleic acid (LA)-derived metabolites. AAs and DHAs included PUFAs, oleoyl ethanolamide (OEA), and lyso-platelet activating factor (PAF). Notably, the urinary levels DHA was increased by approximately 3-fold in COVID-19 patients compared to those in healthy controls.

**TABLE 3:** Urinary lipid profile of COVID-19 patients and healthy subjects.

Lipids	Ratio to IS		Increase ratio (vs. healthy)	*p*-value
	Healthy subjects (Mean ± SEM)	COVID-19 patients (Mean ± SEM)		
Tetranor-PGFM	0.20 ± 0.01	0.44 ± 0.07	2.15	0.007
Tetranor-PGEM	0.87 ± 0.10	4.17 ± 1.25	4.77	0.01
Tetranor-PGDM	0.43 ± 0.05	0.38 ± 0.07	0.88	0.59
20-hydroxy-PGF_2α_	0.09 ± 0.01	0.11 ± 0.03	1.24	0.53
20-hydroxy-PGE_2_	3.38 ± 1.58	1.03 ± 0.32	0.30	0.15
2,3-dinor-8-iso-PGF_2α_	0.36 ± 0.03	0.43 ± 0.16	1.18	0.68
8-iso-PGF_2α_	0.031 ± 0.003	0.035 ± 0.004	1.12	0.48
5-iPF_2α_ –VI	0.15 ± 0.01	0.14 ± 0.01	0.92	0.60
PGF_2α_	0.14 ± 0.02	0.17 ± 0.02	1.16	0.44
8-iso-PGE_2_	0.017 ± 0.004	0.015 ± 0.005	0.86	0.71
PGE_2_	0.060 ± 0.009	0.055 ± 0.009	0.90	0.75
11-dehydro-TXB_2_	0.034 ± 0.003	0.059 ± 0.007	1.76	0.002
15-keto-PGE_2_	0.35 ± 0.13	0.09 ± 0.02	0.26	0.05
LTE_4_	0.019 ± 0.004	0.022 ± 0.005	1.12	0.74
12,13-DiHOME	0.10 ± 0.02	0.10 ± 0.02	0.97	0.94
9,10-DiHOME	2.49 ± 0.87	1.25 ± 0.22	0.50	0.17
Lyso-PAF	0.08 ± 0.04	0.05 ± 0.03	0.64	0.57
9-HODE	0.011 ± 0.001	0.03 ± 0.01	2.80	0.24
OEA	0.16 ± 0.03	0.36 ± 0.16	2.16	0.24
DHA	0.029 ± 0.007	0.09 ± 0.01	3.37	0.002
AA	0.09 ± 0.04	0.23 ± 0.06	2.45	0.07

### Urinary levels of arachidonic acid derived metabolites were significantly increased in coronavirus diseases 2019 patients

Archambault et al. reported that the levels of major proinflammatory mediators such as prostaglandin (PG) E_2_, PGD_2_, thromboxane (TX) B_2_, leukotriene (LT) B_4_, and LTE_4_ in alveolar lavage fluid of patients with severe COVID-19 were higher than those in a control group of healthy subjects (13). In line with these observations, we found a PGE_2_ metabolite, tetranor-PGEM; TXA_2_ metabolite, 11-dehydro-TXB_2_; and a PGF_2α_ metabolite, tetranor-PGFM, were excreted at significantly higher levels in COVID-19 patients than in healthy subjects (Table 3). These significantly changed lipids were all cyclooxygenase (COX) metabolites of arachidonic acids (Supplementary Figure 1).

### Correlation between urinary lipids and plasma biomarkers

Previous reports have proposed the use of serum CRP, D-dimer, procalcitonin, and ferritin as indices for predicting the prognosis of COVID-19. Next, we calculated the co-relation between the detected 21 urinary lipids and plasma biomarkers. We found ten significant correlations between them (Table 4 and Supplementary Table 3). Among these, urinary tetranor-PGEM and ferritin levels in the plasma had the strongest positive correlation (*r* = 0.70). As we showed that urinary tetranor-PGEM levels in patients were significantly higher than those in healthy subjects, urinary tetranor-PGEM levels may be a useful index for estimating ferritin levels in plasma.

**TABLE 4:** Significant correlation values between urinary lipids and plasma biomarkers.

	Plasma biomarkers			
Urinary lipids	CRP	D-dimer	Procalcitonin	Ferritin
Tetranor-PGFM	0.42 (*p* < 0.01)	–	–	–
Tetranor-PGEM	0.65 (*p* < 0.01)	–	–	0.70 (*p* < 0.01)
11-dehydro-TXB_2_	0.28 (*p* = 0.04)	0.44 (*p* < 0.01)	–	–
Tetranor-PGDM	–	–	–	–
20-hydroxy-PGF_2_α	–	–	–	–
20-hydroxy-PGE_2_	–	–	–	–
2,3-dinor-8-iso-PGF_2_α	0.68 (*p* < 0.01)	–	–	–
8-iso-PGF_2_α	–	–	–	–
5-iPF_2_α-VI	–	–	–	–
PGF_2_α	–	–	–	–
8-iso-PGE_2_	–	–	–	–
PGE_2_	0.39 (*p* < 0.01)	–	–	–
15-keto-PGE_2_	–	–	–	–
LTE_4_	0.38 (*p* < 0.01)	0.48 (*p* < 0.01)	–	–
12,13-DiHOME	–	–	–	–
9,10-DiHOME	–	–	–	–
Lyso-PAF	–	–	–	–
9-HODE	–	–	–	–
OEA	–	–	–	–
DHA	0.48 (*p* < 0.01)	–	–	–
AA	–	–	–	–

## Discussion

Although much effort toward COVID-19 diagnostics and treatments has been made, we have not yet managed to control this infectious disease. During the diagnostic procedure, several general biochemical tests can reflect the status of individuals with COVID-19. A previous report showed that the severity and mortality of COVID-19 are associated with elevated levels of serum CRP, ferritin, and D-dimer (17–19). In addition to changes in these blood biomarkers, it is thought that lipid mediators in the urine can also reflect the status of individuals with COVID-19.

Here, we analyzed the urine of 16 patients with mild-to-moderate COVID-19 symptoms in Japan, focusing on changes in fatty acids and lipid metabolites that reflect the inflammatory status of the body. We detected significant increases in the levels of PUFAs and PGE_2_-, PGF_2α –_, and TXA_2_-metabolites in the urine of patients even though their symptoms were not severe. These are sensitive and convenient indices for capturing the disease status of each patient. Archambault et al. suggested the occurrence of lipid storms in the lungs of patients with severe COVID-19 cases where mechanical ventilation was necessary by showing robust production of cyclooxygenase (COX)/lipoxygenase (LOX) metabolites, such as TXB_2_, PGE_2_, 12-hydroxy-heptadecatrenoic acid (HHT), PGD_2_, LTB_4_, LTE_4_, 12-hydroxy-eicosatetraenoic acid (HETE), and 15-HETE in their BALFs (13). Notably, some of these lipid metabolites were also detected in the urine of mildly ill patients. These mediators may be crucial for the onset and progression of the disease. At the same time, these can be sensitive indices to evaluate the inflammatory status of each patient. Since the PUFAs and lipid metabolites detected in the urine of COVID-19 patients have also been reported to be found in several types of diseases (20, 21), these lipid profiles are likely not specific for COVID-19 and respiratory symptoms. However, these can be useful indices of disease status and severity in patients after diagnosis by RT-PCR. This can also be useful for predicting the therapeutic efficacy.

Our data showing an increase in the level of PGs suggested the activation of phospholipase A_2_ (PLA_2_) and/or COX in patients. Several studies have reported that some viral infections stimulate PLA_2_ and/or COX activation (22). SARS-CoV-2 invasion and replication are likely to damage respiratory cells directly and indirectly, leading to PLA_2_ activation. We also detected several types of COX metabolites in urine. We believe that some COX metabolites are increased during cytokine storms in patients. Indeed, the levels of the major cytokine IL-1β were elevated in the plasma of some COVID-19 patients (23) and IL-1β is a potent inducer of COX-2 expression (24).

Notably, we found a significant positive correlation between urinary tetranor-PGEM and plasma ferritin levels. Elevated ferritin levels are a hallmark of macrophage activation syndrome (MAS) (25). MAS may contribute to aspects of the cytokine storm and the hypercoagulable state observed in COVID-19. As previously reported in a combined analysis of 653 COVID-19 patients across two studies, patients with severe symptoms had higher serum ferritin levels than non-severe symptoms with an average difference of 408 ng/mL (95% CI 311–505), and non-surviving patients had higher ferritin levels than surviving patients with an average difference of 760 ng/mL (95% CI 561–959) ^25^. As it is well known that macrophages can produce PGE_2_ in activation (26), it is reasonable that tetranor-PGEM, the metabolite of PGE_2_ can be more detected in MAS. Thus, urinary tetranor-PGEM levels, as well as plasma ferritin levels, can be used as severity indices reflecting MAS in COVID-19. Considering our result that urinary PGE_2_ level was not different between COVID-19 patients and healthy subjects, we suppose that metabolization of PGE_2_ into tetranor-PGEM is promoted in urine of COVID-19 patient.

Urine is often used as a biomaterial for biomarker discovery in human diseases because of its ease of accessibility and non-invasive sampling. Since some lipid mediators are metabolized within minutes in tissues and blood and excreted in urine, urine has another advantage in that these metabolites can be stably detected. In addition, blood test is available in the clinics and hospitals, while the urine can be easily and safely sampled even at home. By developing urine markers and at-home-clinical-diagnosis kits, we could contribute to avoid unnecessary contact with positive patients and lower the dissemination of COVID-19 infection. In contrast, urine samples contain matrixes which can interfere the measurement (27). There is a need to develop a urine extraction method that will allow accurate measurement of lipid concentrations in urine in various condition. In addition, although there are few studies showing the existence of virus particles in urine, Peng et al. reported that SARS-CoV-2 RNA was detected in the urine of patients (28). For biosafety reasons, it is imperative to pay attention to how the patients’ urine samples are handled. In the present study, 50% methanol was added to neutralize the virus particles and maintain excreted lipids. Therefore, urine collection and storage methods that enable safe and stable detection should be considered.

This study had some limitations. First, the number of samples collected was small. The coverage of population and representativeness was limited when sex, age, nationality, and symptom severity were considered. More sample applications in future studies will eliminate the possibility of sample bias. Second, the current patients were not subjected to drug treatments, especially to NSAIDs, and further study is needed to reveal the types of drugs that influence the production and excretion of lipid mediators in clinical settings. Third, the samples analyzed in this study were collected between June and October 2020. Thus, the SARS-CoV-2 has undergone considerable mutation. With mutations, the symptoms of COVID-19 may have changed. Thus, it is possible that the profile of urinary lipid metabolites has changed. Finally, the methodology of analyzing urinary lipids by mass spectrometry has some limits including quantification, extraction efficiency and matrix effect. Thus, we need to validate the experimental condition when apply to the medical practice.

Our data indicated that the urinary lipid profile of COVID-19 patients is characterized by (1) an increase in levels of DHA; (2) an increase in COX metabolite levels, notably the level of PGE_2_ metabolite tetranor-PGEM and TXA_2_ metabolite 11-dehydro-TXB_2_; and (3) a correlation between urinary tetranor-PGEM and plasma ferritin. We hope that these observations will contribute to the development of safer and more efficacious treatments for COVID-19.

## Data availability statement

The original contributions presented in this study are included in the article/Supplementary material, further inquiries can be directed to the corresponding author.

## Ethics statement

The studies involving human participants were reviewed and approved by the Ethics Committee of the International University of Health and Welfare (20Nr-001) and the University of Tokyo (No. 10586-1). Written informed consent for participation was not required for this study in accordance with the national legislation and the institutional requirements.

## Author contributions

MK, TN, and TS performed the experiments. MK, TN, KK, TS, and TM analyzed the data. All authors designed the research and wrote the manuscript.

## Abbreviations

AA: arachidonic acid
BALF: bronchoalveolar lavage fluid
COVID-19: coronavirus diseases 2019
COX: cyclooxygenase
CRP: c-reactive protein
DHA: docosahexaenoic acid
DiHOME: dihydroxy-octadecenoic acid
EPA: eicosapentaenoic acid
EpOME: epoxy-octadecamonoenoic acid
HETE: hydroxy-eicosatetraenoic acid
HHT: hydroxy-heptadecatrienoic acid
HODE: hydroxy-octadecadienoic acid
HpETE: hydroperoxy-eicosatetraenoic acid
HpODE: hydroperoxyl-octadecadienoic acid
IL: interleukin
IS: internal standard
LA: linoleic acid
LOX: lipoxygenase
LT: leukotriene
MAS: macrophage activation syndrome
OEA: oleoyl ethanolamide
PAF: platelet-activating factor
PG: prostaglandin
PLA_2_: phospholipase A_2_
PUFA: poly unsaturated fatty acid
RT-PCR: real-time reverse transcription polymerase chain reaction
SARS-CoV-2: severe acute respiratory syndrome coronavirus 2
TX: thromboxane.
