# Supplementary material for: Urinary lipid profile of patients with coronavirus diseases 2019
Source: Front Med (Lausanne). 2022 Sep 26;9:941563. doi: 10.3389/fmed.2022.941563 (PMC9548532; doi:10.3389/fmed.2022.941563)
Supplement: Supplementary file 1 [file Data_Sheet_1.PDF]

Supplementary Material

1 Supplementary Figures and Tables

1.1 Supplementary Figures

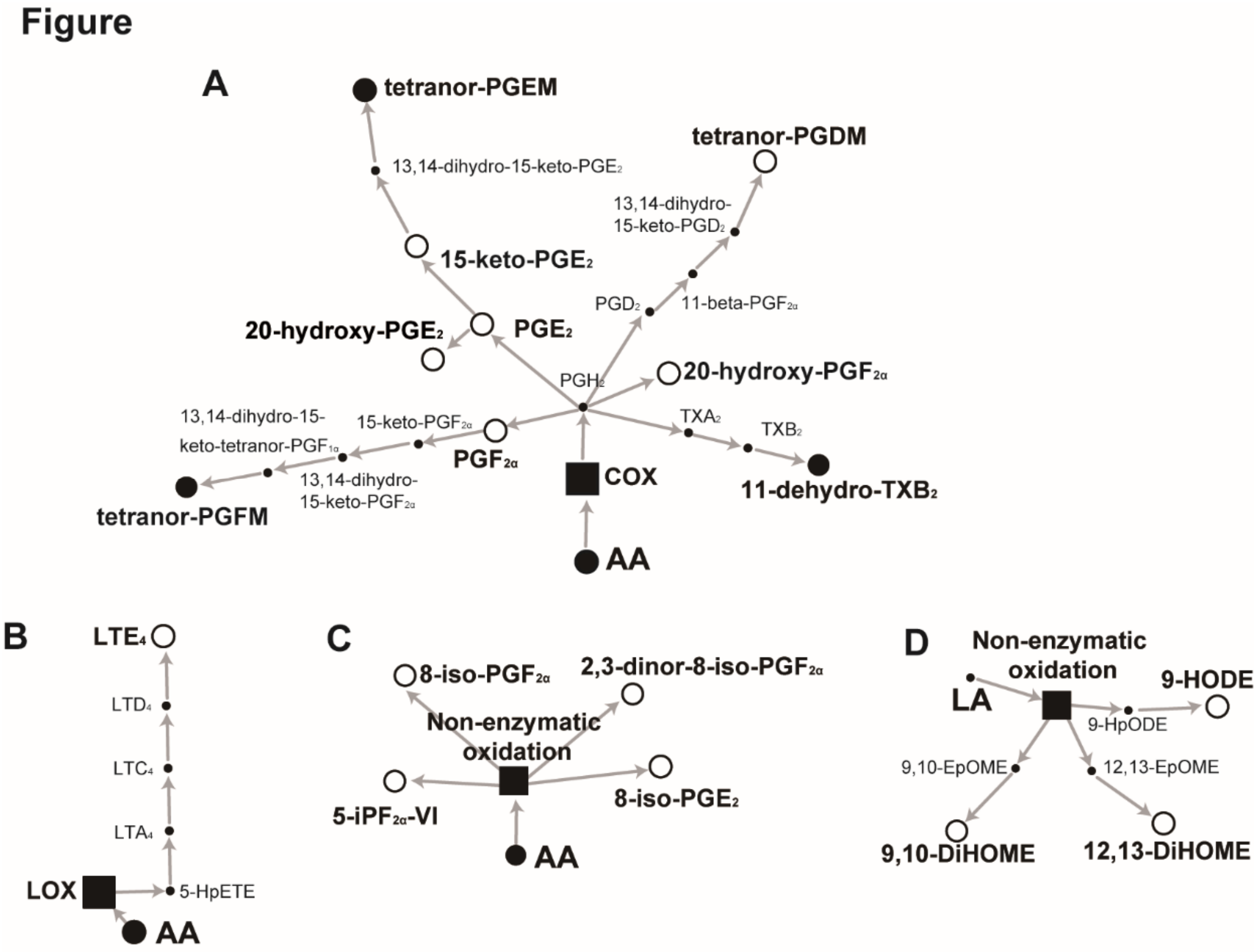

Supplementary Figure 1. Metabolic pathway of AA and LA

AA (A, B and C) and LA (D) metabolic pathway classified by COX, LOX or non-enzymatic oxidation. A black dot indicates analyzed but not detected lipid metabolite in urine of healthy and patient. A black open circle indicates non-significantly changed metabolite. A black filled circle indicates significantly changed metabolite in patient compared to healthy.

1.2 Supplementary Tables

Supplementary Table 1. Internal standard

Target (ng/ml)	Retention time	Precursor	Product
tetranor-PGEM-d_6 (25)	3.56	333.2	315.2
6-keto-Prostaglandin $F_{1\alpha}$-d_4 (25)	8.23	373.2	249.1
Thromboxane B_2-d_4 (25)	10.08	373.2	199.1
Prostaglandin $F_{2\alpha}$-d_4 (25)	10.84	357.2	197.2
Prostaglandin E_2-d_4 (25)	11.25	355.2	275.2
Prostaglandin D_2-d_4 (25)	11.65	355.2	275.2
Leukotriene C_4-d_5 (25)	13.23	631.4	308.2
Leukotriene B_4-d_4 (25)	14.64	339.2	197.1
5(S) HETE-d_8 (25)	17.84	327.2	116.1
12(S) HETE-d_8 (25)	17.74	327.2	184.3
15(S) HETE-d_8 (25)	17.38	327.2	226.2
PAF C-16-d_4 (25)	17.49	572.4	59.1
Oleoyl Ethanolamide-d_4 (0.5)	19.67	330.2	66.1
Eicosapentaenoic Acid-d_5 (500)	19.90	306.2	262.2

Docosahexaenoic Acid-d_5 (500)	20.65	332.2	288.2
Arachidonic Acid-d_8 (500)	20.77	311.2	311.2

Supplementary Table 2. Complete profile of metabolites measured in the current study.

	Healthy subjects															
**No.**	**1**	**2**	**3**	**4**	**5**	**6**	**7**	**8**	**9**	**10**	**11**	**12**	**13**	**14**	**15**	**16**
**Symptoms**	None	None	None	None	None	None	None	None	None	None	None	None	None	None	None	None
**Lipids**																
**tetranor-PGFM**	0.309	0.222	0.207	0.255	0.162	0.295	0.241	0.177	0.203	0.143	0.232	0.121	0.183	0.105	0.276	0.136
**tetranor-PGEM**	0.680	1.441	0.967	0.633	0.561	0.537	1.545	0.876	1.220	0.931	1.849	0.356	0.718	0.519	0.348	0.801
**tetranor-PGDM**	0.618	0.126	0.486	0.449	1.129	0.508	0.491	0.305	0.330	0.412	0.238	0.109	0.509	0.315	0.417	0.564
**20-hydroxy-PGF2α**	0.065	0.316	0.114	0.055	0.055	0.019	ND	0.111	0.061	0.073	0.166	0.116	0.095	0.074	0.063	0.037
**20-hydroxy-PGE2**	5.296	2.675	0.688	0.793	0.221	0.857	25.826	2.408	1.158	0.399	1.234	0.297	7.470	0.313	3.619	0.884
**2,3-dinor-8-iso-PGF2α**	0.301	0.266	0.263	0.408	0.578	0.326	0.411	0.223	0.811	0.191	0.354	0.437	0.369	0.144	0.382	0.407
**8-iso-PGF2α**	0.020	0.042	0.018	0.022	0.044	0.032	0.035	0.028	0.049	0.021	0.044	0.038	0.015	0.016	0.012	0.069
**5-iPF2a-VI**	0.111	0.214	0.079	0.162	0.222	0.073	0.230	0.126	0.296	0.108	0.221	0.215	0.089	0.076	0.105	0.224
**PGF2α**	0.074	0.431	0.076	0.094	0.143	0.077	0.170	0.222	0.188	0.064	0.140	0.194	0.111	0.097	0.098	0.168
**8-iso-PGE2**	0.034	0.015	0.006	0.013	0.010	ND	0.059	0.001	0.017	0.009	ND	0.029	0.022	0.004	0.018	0.005
**PGE2**	0.034	0.112	0.076	0.031	0.042	0.016	0.024	0.030	0.043	0.045	0.123	0.256	0.027	0.031	0.046	0.030
**11-dehydro-TXB2**	0.057	0.019	0.042	0.050	0.042	0.027	0.037	0.016	0.045	0.022	0.026	0.029	0.044	0.017	0.037	0.028
**15-keto-PGE2**	1.098	0.003	0.072	0.218	0.003	0.144	0.030	0.032	0.241	0.037	1.293	0.778	0.003	0.075	1.599	0.116
**LTE4**	0.008	0.006	0.049	0.027	0.074	0.007	0.016	0.010	0.011	0.011	0.013	0.006	0.022	0.006	0.010	0.033
**12,13-DiHOME**	0.043	0.023	0.047	0.133	0.077	0.050	0.161	0.030	0.060	0.040	0.044	0.187	0.223	0.129	0.381	0.023
**9,10-DiHOME**	0.396	0.359	0.277	0.367	0.550	1.524	8.928	1.154	0.391	0.831	1.075	2.026	3.197	7.226	11.335	0.259
**Lyso-PAF**	0.063	0.002	0.697	0.026	0.053	0.042	0.047	0.007	0.025	0.017	0.010	0.010	0.007	0.023	0.367	0.017
**9-HODE**	0.008	0.004	0.008	0.028	0.012	0.007	0.008	0.003	0.011	0.004	0.014	0.022	0.019	0.013	0.011	0.009
**OEA**	0.104	0.040	0.256	0.497	0.474	0.151	0.056	0.084	0.261	0.101	0.210	0.067	0.056	0.058	0.170	0.079
**DHA**	0.053	0.014	0.127	0.028	0.034	0.012	0.033	0.011	0.015	0.013	0.012	0.024	0.010	0.017	0.042	0.015
**AA**	0.055	0.045	0.713	0.078	0.076	0.039	0.031	0.016	0.072	0.043	0.032	0.045	0.053	0.060	0.095	0.043

ND: not detected

	Patients with COVID-19															
**No.**	**1**	**2**	**3**	**4**	**5**	**6**	**7**	**8**	**9**	**10**	**11**	**12**	**13**	**14**	**15**	**16**
**Symptoms**	Fever and cough	Fever, cough and taste disorder	Fever, cough and fatigue	Fever, cough, headache and diarrhea	Fever, cough and fatigue	Fever, ouch, headche and chest pain	Fever	Fever, cough, taste disorder, smell disoeder and fatigue	Cough and fatigue	Fever, cough, fatigue, diarrhea and vomiting	Fever	Fever, dyspnea and chest pain	Fever, cough and fatigue	Fever, cough, dyspnea and diarrhea	Fever and headache	Fever and cough
**Lipids**																
**tetranor-PGFM**	1.252	0.178	0.324	0.288	0.403	0.191	0.500	0.568	0.123	1.046	0.315	0.659	0.515	0.151	0.232	0.293
**tetranor-PGEM**	21.793	1.494	6.027	2.289	2.951	1.207	3.398	2.900	0.869	3.719	2.363	5.874	3.174	1.409	1.116	6.156
**tetranor-PGDM**	0.068	0.291	0.461	0.461	0.340	0.304	0.603	1.293	0.079	0.551	0.240	0.714	0.376	0.099	0.277	0.018
**20-hydroxy-PGF2α**	0.493	0.107	0.073	0.103	0.071	0.092	0.037	0.142	0.083	0.029	0.130	0.333	0.038	0.069	0.021	0.058
**20-hydroxy-PGE2**	5.383	1.314	0.687	0.335	0.351	0.174	0.479	0.890	0.954	0.236	1.995	1.568	0.132	1.480	0.122	0.388
**2,3-dinor-8-iso-PGF2α**	2.934	0.149	0.196	0.232	0.260	0.233	0.279	0.424	0.189	0.267	0.386	0.383	0.366	0.211	0.306	0.168

**8-iso-PGF2α**	0.063	0.024	0.023	0.012	0.033	0.027	0.036	0.060	0.066	0.032	0.044	0.040	0.028	0.040	0.026	0.013
**5-iPF2a-VI**	0.180	0.165	0.064	0.103	0.153	0.121	0.177	0.229	0.227	0.114	0.159	0.152	0.200	0.191	0.091	0.039
**PGF2α**	0.164	0.231	0.095	0.101	0.101	0.287	0.112	0.325	0.312	0.253	0.137	0.104	0.211	0.179	0.103	0.027
**8-iso-PGE2**	0.037	0.006	0.007	0.010	0.011	0.006	0.009	0.016	0.084	0.007	0.008	0.012	0.009	0.002	0.008	0.004
**PGE2**	0.139	0.038	0.061	0.022	0.013	0.098	0.037	0.084	0.115	0.033	0.031	0.052	0.027	0.066	0.043	0.016
**11-dehydro-TXB2**	0.070	0.021	0.032	0.029	0.070	0.051	0.117	0.093	0.057	0.048	0.088	0.052	0.047	0.030	0.039	0.108
**15-keto-PGE2**	0.015	0.019	0.106	0.065	0.015	0.168	0.094	0.026	0.023	0.364	0.028	0.131	0.245	0.018	0.222	0.003
**LTE4**	0.059	0.006	0.005	0.008	0.020	0.020	0.012	0.040	0.014	0.012	0.091	0.018	0.016	0.008	0.013	0.005
**12,13-DiHOME**	0.048	0.011	0.045	0.057	0.160	0.083	0.095	0.280	0.072	0.054	0.315	0.076	0.106	0.038	0.053	0.120
**9,10-DiHOME**	0.681	0.063	0.871	2.102	2.651	1.429	0.991	2.796	1.348	0.291	2.449	1.838	0.533	0.468	0.329	1.264
**Lyso-PAF**	0.013	0.005	0.012	0.004	0.008	0.007	0.486	0.038	0.006	0.041	0.012	0.095	0.147	0.005	0.023	0.015
**9-HODE**	0.032	0.002	0.015	0.014	0.018	0.024	0.016	0.287	0.007	0.013	0.010	0.028	0.022	0.003	0.007	0.009
**OEA**	0.070	0.020	0.772	0.188	0.180	0.051	0.135	2.543	0.076	0.190	0.077	0.988	0.215	0.033	0.191	0.030
**DHA**	0.272	0.005	0.122	0.020	0.018	0.101	0.116	0.068	0.034	0.064	0.208	0.072	0.205	0.013	0.110	0.123
**AA**	0.646	0.020	0.178	0.094	0.084	0.154	0.206	0.199	0.062	0.185	0.046	0.221	0.741	0.037	0.719	0.090

Supplementary Table 3. Non-significant correlation values between urinary lipids and plasma biomarkers

Lipids	CRP	D-dimer	Procalcitonin	Ferritin
tetranor-PGFM	-	0.07	-0.16	0.12
tetranor-PGEM	-	0.19	0.05	-
11-dehydro-TXB_2	-	-	0.11	-0.05
tetranor-PGDM	-0.08	-0.20	-0.09	-0.06
20-hydroxy-$PGF_{2\alpha}$	0.15	0.02	-0.17	-0.08
20-hydroxy-PGE_2	-0.12	-0.03	-0.07	0.07
2,3-dinor-8-iso-$PGF_{2\alpha}$	-	0.01	-0.17	-0.15
8-iso-$PGF_{2\alpha}$	0.16	0.00	0.17	-0.01
5-$iPF_{2\alpha}$-VI	-0.10	-0.08	-0.20	0.11
$PGF_{2\alpha}$	0.06	-0.03	0.05	-0.27
8-iso-PGE_2	0.11	-0.22	-0.14	-0.15
PGE_2	-	-0.11	0.16	0.02
15-keto-PGE_2	-0.09	-0.06	-0.13	-0.12
LTE_4	-	-	-0.01	-0.20
12,13-DiHOME	-0.12	0.03	-0.06	0.11
9,10-DiHOME	-0.09	0.03	-0.02	0.02
Lyso-PAF	0.09	0.20	-0.01	0.01

9-HODE	0.04	-0.05	-0.02	-0.03
OEA	-0.12	-0.13	-0.20	0.02
DHA	-	0.16	0.10	-0.12
AA	0.25	-0.13	0.01	-0.21
